# Ethical and practical considerations related to data sharing when collecting patient-reported outcomes in care-based child health research

**DOI:** 10.1007/s11136-023-03393-2

**Published:** 2023-03-31

**Authors:** Shelley Vanderhout, Beth K. Potter, Maureen Smith, Nancy J. Butcher, Jordan Vaters, Pranesh Chakraborty, John Adams, Michal Inbar-Feigenberg, Martin Offringa, Kathy Speechley, Yannis Trakadis, Ariella Binik

**Affiliations:** 1 School of Epidemiology and Public Health, University of Ottawa, Ottawa, Canada; 2 Patient Partner, Canadian Organization for Rare Disorders, Toronto, Canada; 3 Patient Partner, Canadian PKU and Allied Disorders, Inc., Toronto, Canada; 4 The Hospital for Sick Children Research Institute, Peter Gilgan Centre for Research & Learning, Toronto, Canada; 5 Department of Psychiatry, Faculty of Medicine, University of Toronto, Toronto, Canada; 6 Newborn Screening Ontario, Children’s Hospital of Eastern Ontario, Ottawa, Canada; 7 Department of Philosophy, McMaster University, University Hall, 1280 Main St W, Hamilton, ON L8S 4K1 Canada; 8 Department of Paediatrics, University of Western Ontario, London, Canada; 9 The Hospital for Sick Children, Toronto, ON Canada; 10 Department of Human Genetics, McGill University, Montreal, Canada

**Keywords:** Patient-reported outcomes, Children, Research ethics, Data sharing

## Abstract

**Purpose:**

The collection and use of patient reported outcomes (PROs) in care-based child health research raises challenging ethical and logistical questions. This paper offers an analysis of two questions related to PROs in child health research: (1) Is it ethically obligatory, desirable or preferable to share PRO data collected for research with children, families, and health care providers? And if so, (2) What are the characteristics of a model best suited to guide the collection, monitoring, and sharing of these data?

**Methods:**

A multidisciplinary team of researchers, providers, patient and family partners, and ethicists examined the literature and identified a need for focus on PRO sharing in pediatric care-based research. We constructed and analyzed three models for managing pediatric PRO data in care-based research, drawing on ethical principles, logistics, and opportunities to engage with children and families.

**Results:**

We argue that it is preferable to share pediatric PRO data with providers, but to manage expectations and balance the risks and benefits of research, this requires a justifiable data sharing model. We argue that a successful PRO data sharing model will allow children and families to have access to and control over their own PRO data and be engaged in decision-making around how PROs collected for research may be integrated into care, but require support from providers.

**Conclusion:**

We propose a PRO data sharing model that can be used across diverse research settings and contributes to improved transparency, communication, and patient-centered care and research.

## Plain English summary

Patient reported outcomes (PROs) are used in both health care and research to capture aspects of patient health such as symptom severity, quality of life, and day-to-day functioning. In research studies based in children’s health care settings, PROs are sometimes collected but not always shared with children, families, or providers. As a team of researchers, providers, patient partners, and ethicists, we considered ethical and logistical aspects of sharing PROs collected for care-based research, with a focus on supporting child and family-centred research and care. We analyze three models that could support handling PROs, considering logistics, patient and family perspectives, and ethical principles such as privacy and respect for persons. We suggest that when sharing PRO data in care-based research, children and families may benefit from access to and control over their own PRO data and being engaged in decision-making around how PROs collected for research may be integrated into care, but this requires support from researchers and providers.

## Background

Patient-reported outcomes (PROs) are defined by the International Society for Quality of Life Research [1] as “measurement[s] of any aspect of a patient’s health that comes directly from the patient, without interpretation of the patient’s response by a physician or anyone else.” PROs are used to capture subjective health measures including symptom severity, health-related quality of life, functionality, and health behaviours [2]. Evidence suggests that PROs contribute to improved communication, shared decision-making, and patients’ understanding of their health [3–7]. In care-based research— defined as research that takes place during clinical encounters with a patient’s usual care provider, including pragmatic randomized controlled trials, registry trials, and learning health care systems [8]—collecting PROs can help gain insight into patient perspectives and generate evidence for patient-centred practices in health care [9–11]. In pediatric settings, PRO measurement tools have been adapted to suit children of different ages [12, 13]. If a child is too young or otherwise not able to report outcomes themselves, parents or caregivers may report PROs on children’s behalf [14], but evidence suggests that children as young as eight years old have the ability to complete quality of life measures [32]. Although caregivers often act as mediators between providers and children and can provide insight to children’s well-being and daily functioning, child-reported PROs are increasingly being used in routine care and research [33] because subjective measures such as emotional state, pain, and quality of life are difficult to capture through a proxy without bias [15, 16].

While the benefits of collecting PROs in both health care and research are increasingly recognized [9–11], questions remain about the collection and use of PRO data, including whether and when PRO data collected in health care may be used for research purposes, whether there is an obligation to share PRO data collected for research with the health care team, whether sharing PRO data is ethically permissible or desirable, and how patients and families should be involved in decisions about PRO data collection and sharing. These questions are particularly pressing when collecting PRO data in pediatric research due to children’s limited ability to protect and promote their own interests. Commentators have pointed to a need for guidance regarding PRO reporting and response during research [17, 18] and analysis of ethical questions concerning PROs [19].

As a team of researchers, providers, patient partners, and ethicists, we contribute to these gaps by analyzing two questions: (1) Is it ethically obligatory or preferable for researchers to share PRO data collected for research purposes with children, families, health care teams? And if so, (2) What are the characteristics of a model best suited to guide the collection, monitoring, and sharing of pediatric PRO data? Incorporating the different perspectives of the authorship team, we examine three potential models for collecting and sharing PRO data in pediatric research and endorse a new PRO data sharing model.

## Existing guidance

Some guidance exists for the design, conduct, and reporting of randomized controlled trials that collect PROs in adults [20, 21]. There has been some discussion about ethical collection and use of PROs in clinical care, focused on the integrating PRO data collected during research into patients’ electronic health records, with options ranging from no disclosure to a detailed informed consent process including an opt-in requirement [22–24]. A set of ethical guidelines for research including PROs has recently been published [25], but they do not specify *how* research PROs can be integrated into care [25]. In pediatrics, limited attention has been paid to ethical questions surrounding PRO collection and data sharing in cohort or registry studies where consent and assent, child and family preferences, and clinical practice guidelines may change over time (i.e., in accordance with a child’s age or capacity to provide consent). There is no published best practice on how to account for child and family perspectives, values, and preferences around these issues. We build on these gaps below.

## Ethical obligations to share PRO data

One concern is whether there is an ethical obligation to monitor, flag, review, share, or respond to clinically actionable PROs and if so, who should be responsible for monitoring and acting on such responses. Clinically actionable data is not a clear cut category, but can include measures that capture a person’s mental health and reveal assessments such as severe depression. Several approaches have been considered for managing such responses: researchers could be trained to review and flag concerning responses [26], patients could be asked to self-report their own concerns through a help line, an automated alert system for concerning PRO responses could be established [27], or participants could provide consent for researchers to document PROs in medical records and notify providers if concerning responses occur, which shifts the responsibility of addressing responses to providers [22, 28].

Additional work is needed to clarify what is meant by clinically actionable or concerning responses, whether the research team’s duties can be fulfilled by flagging responses for clinical follow-up, which (if any) of the suggested responses above is most advantageous, and how follow-up should proceed. Despite differing suggestions, one broad point of consensus is that researchers must make plans to monitor and address potentially concerning and clinically actionable responses in PROs. This is supported by guidelines for collecting PROs in research [25] and by the ethical principle of beneficence, which holds that researchers must promote the interests of research participants and guarantee a reasonable balance between the risks and benefits of research. Ethically and logistically permissible approaches to monitoring and addressing concerning and clinically actionable responses in PRO data is an important issue, but one we leave for future analysis.

For the moment, we focus on PROs that will not meet thresholds for obligatory follow-up, which account for a large quantity of PRO data and can offer insights about patient wellbeing (e.g., pain levels, coping techniques, satisfaction with new interventions, unforeseen challenges or benefits of a new treatment mode). It can be advantageous and preferable to share PRO data that do not raise concerning responses with children, families, and their providers, but these groups are likely to have different preferences concerning data sharing. We address this below by constructing and examining three different approaches for collecting and sharing non-clinically concerning PRO data.

## Approaches for collection and sharing of research PROs

We reviewed existing literature [22–25], much of which focuses on adult populations and integrating PROs into electronic health records, to develop and examine three models (Table 1) for handling PROs collected for pediatric care-based research. We adapted existing approaches [22] to address our questions of how to manage PRO data collected for research purposes with providers and with research participants in pediatric care-based research. While data sharing in the reverse direction (i.e., PROs collected during clinical care that could be shared with researchers) is an important issue related to our analysis, we have limited the scope of this paper to address the potential for sharing PROs initially collected for research, a separable problem that has received less attention. Each of the models require children and families to make informed decisions, some of which are complex and may necessitate age-appropriate dialogue, and a child’s capacity to consent or assent is implicated in these decisions. Ethics guidelines generally require children’s assent (agreement) or consent in addition to authorization from a parent or guardian [29–31]. Assent and consent requirements may vary depending on a child’s age, jurisdiction, or capacity levels, but families, providers, and researchers should take steps to include children in decision making by offering adequate information about data sharing options and presenting it appropriately for a child’s level of maturity [30]. Seeking assent in these ways helps to contribute to children’s developing autonomy [29]. In addition, a child’s dissent for participation in data sharing should be respected. This is consistent with ethics guidelines and commentary, which suggests that dissent to participate may only be overruled in limited circumstances involving the need for treatment unavailable outside the context of a research study [29–31].

**Table 1:** Models for handling PROs collected in care-based research

Model	Description
1: Closed	- PROs collected solely for research purposes - No obligation or expectation that “low-risk” study PRO data be shared between the research and clinical teams - Children, families, or providers have no access to PRO data (aside from individual, specific requests to an investigator for its release)
2: Child and family decide whether to share data	- Children and families are provided access to their own PRO data and have the option to present them to providers - During the consent process for research participation, children and families are informed that, “as a default,” PROs they provide for research will not be communicated to providers or documented in medical records, but that they may choose to share these data with their providers during clinical encounters - Research team may advise children and families about the “added value” of providers having access to PRO data and the option to share them, but ultimately the decision is left to the child and family
3: Child and family option to share data with clinician opt-in/opt-out	- Children, families, and providers are given the opportunity to provide consent to share and receive (a subset of) PRO data, respectively - Providers can opt in or out of reviewing, interpreting, and integrating PROs into care - Offers the potential for PROs collected for research to be integrated into clinical care

## Model analysis

To analyze the models, we draw on existing guidance concerning the collection and use of PROs in clinical care [3, 6], ethical principles such as respect for persons, beneficence, and privacy, logistical constraints, and child and family engagement (i.e., including children and families in the design and conduct of research). An overview of our analysis criteria is presented in Table 2 and outcomes in Table 3.

**Table 2:** Ethical and practical criteria for evaluating PRO data handling models

Criterion	Description
Respect for persons	- Respect for persons is a moral principle giving rise to the moral obligation to respect the self-determination of individuals and also to protect those with limited or developing autonomy [30, 44] - This principle gives rise to the moral duty to provide sufficient information and to seek an individual’s informed consent to enroll as a research participant [30, 44] - When research participants such as young children cannot provide informed consent, this principle can be upheld, in part, by obtaining consent from a parent or legally authorized representative and also by seeking children’s assent, when appropriate [31, 45]
Beneficence	- Beneficence is a moral principle that promotes individual well-being - It gives rise to moral obligations to eliminate unnecessary risks, minimize the risks of necessary research procedures, and to ensure a reasonable balance between the potential benefits and risks of a study [30, 44, 45]
Privacy and confidentiality	- Respecting the rights and welfare of individuals requires that personal information gathered in research be treated confidentially [30] - Privacy rights address freedom for interference by others and the right to control one’s own personal information [30] - Protecting privacy requires close scrutiny of identifiable information and careful attention to potential harms that may result from the collection, use, or disclosure of this information [30] - One way to respect privacy is to provide the opportunity for individuals to consent to or withhold consent for the sharing or disclosure of information [30]
Avoiding the therapeutic misconception	- The therapeutic misconception refers to a research participant’s mistaken belief that all aspects of a study aim to benefit them therapeutically [30, 46] - In this situation, participants may not fully appreciate the difference between clinical care and research participation, the ways in which research and care may be in tension, and that the primary goal of a study is to generate knowledge [30] - Promoting clarity and understanding, which involves avoiding the therapeutic misconception, should be understood as a moral obligation of research
Child and family engagement	- Engaging children and families in discussions about PROs and how their use will inform policy or practice outcomes helps to ensure that relevant perspectives are considered [10, 47, 48] and helps to account for discrepancies in perceptions of health and health changes - Child and family engagement also contributes to the promotion of respect for person, the avoidance of the therapeutic misconception, and to transparency and clarity in research and clinical expectations
Logistics	- Arrangements for sharing PRO data collected for research with the clinical team should follow a clear procedure and must also be feasible for both the research and clinical teams

**Table 3:** Analysis of PRO data handling models

	Model 1: Closed	Model 2: Child and family decision to share data	Model 3: Child and family option to share data with clinician opt-in/opt-out
Respect for persons	- PRO data cannot be incorporated into health care - No timely response to concerning PRO response provided by child’s care team - Least family and child-centred approach	- Care more child-centred if PROs are incorporated - Potential for misinterpretation of results if children and families or providers are not equipped to interpret PROs	- Care more child-centred if PROs are incorporated - Child’s own provider addresses PRO responses
Beneficence	- May not reflect child or family expectations, which may compromise child welfare	- Contributes to the need to develop a system for data sharing and storage for which health risk minimisation considerations would need to be considered carefully	- May result in challenges in provider and child/family relationship if one party consents to share data but the other opts out
Privacy and confidentiality	- PRO data kept private, but not readily accessible to children and families	- Children and families control their own PRO data	- Children and families control their own PRO data - Potential for negative impact on care dynamic if only one party consents to share data
Avoiding the therapeutic misconception	- May help lower the risk; during consent, children and families would be notified that all data are collected for research purposes only, and will not be shared beyond the research team	- Unlikely to prevent and may exacerbate therapeutic misconception due to reliance on children and families to decide to share data (that they may believe has been collected to share with clinical team to maximize child benefit)	- Requires opt-in by multiple stakeholders for different purposes (e.g., research versus care) and necessitates a high degree of communication between children and families, researchers, and clinicians - Contributes to clarification about the purposes of research procedures versus care interventions
Child and family engagement	- Limited opportunities for engagement	- Children and families have the opportunity to integrate PROs and research participation into care, but bear the full responsibility for this to take place	- Children, families, and providers partner to discuss, record, and incorporate PROs into care
Logistics	- Researchers assume limited or no responsibilities to establish communication with providers, data sharing agreements and systems	- Onus is on children and families to interpret and integrate PROs into their care; researchers provide additional support	- Minimized potential for duplicated data collection - Burden on researchers to inform children and families about data sharing process, establish communication with children and families, providers - Providers may require additional training to evaluate, interpret and respond to PROs

### Model 1: closed

In a closed model, PRO data are collected exclusively for research purposes with no expectation or opportunity to share with providers, children, and families. This is similar to what Snyder et al. [22] describe in the context of documenting PRO data in electronic health records as part of clinical care, but in our case addresses PRO data collected for research. One advantage of this model is that it requires limited data-sharing pathways between researchers, children, families, and providers, which may save time and resources [22]. This model may also be appropriate for PROs that are developed specifically for research and have limited clinical actionability, or in situations where a PRO is being used for research in a population for whom it has not been demonstrated to be valid or reliable. In these settings, it may be appropriate not to share data with children, families, or providers, because their clinical implications may be unclear.

Model 1 complicates promoting the principle of respect for persons. When PROs are collected for research rather than during care, children, families, and providers may be less familiar with their purpose and interpretation without guidance [10]. Uncertainty about the purpose of PROs may contribute to misinterpretation of their value [32]. When PROs are collected for research, inconsistencies have been reported in their explained purpose to potential participants and their families [33]. This may give rise to a common misapprehension referred to as the therapeutic misconception, understood as the mistaken belief that all research procedures, including collecting PROs, are in the direct interests of participants [45]. Within some clinical populations (e.g., rare diseases), providers are also often researchers, which can blur the distinction between care and research. On the basis of this belief, children or families may inadvertently rely on PRO measures collected only for research to communicate symptoms or issues not addressed in other clinical encounters [6, 17]. A closed model may not best reflect participant expectations and may not mitigate the therapeutic misconception especially for children, who may not have been provided an accessible, age-appropriate way to understand their own rights and others’ responsibilities in health care. This model also raises important legal and practical questions about ownership of the PRO data collected, which we do not address here.

In Model 1, children and families are asked to provide consent for the collection of PRO data for research, not for sharing these data with providers. In this model, data sharing outside of research does not occur and consequently, the information provided during informed consent may focus mainly on issues of protection of privacy and confidentiality. While this appears to support children’s privacy, the model does not promote children’s and their families’ rights to control their own data, which is also central to respect for privacy. There is also limited room in this model for children’s changing capacity to decide how their information may be shared with their health care team. In this model, there is no opportunity for child and family engagement in decisions about data sharing, which limits the possibility of taking child and family preferences into consideration.

Model 1 is not consistent with often expressed patient preferences to receive their own PRO data once it has been collected and interpreted [4, 34]. Patients have indicated that they prefer to be in control of their own health data regardless of whom they ultimately are shared with [4]. Some patients have expressed that they want providers to have access to their PRO data, and do not have concerns about who has access to them provided that they are used for patient care, provider training, or research purposes [34]. However, there may be circumstances where patients and providers feel this access is less necessary or appropriate. Regardless, providing an opportunity to consider individual preferences assumes the most patient-centred approach. Many health care systems have moved towards providing child and family access to electronic health records to facilitate open communication and autonomy [35], including giving children with capacity to consent full control over their health information [36]. A similar approach could be adopted for PROs collected for research to accommodate varied child and family preferences.

Despite these disadvantages, Model 1 is not atypical. Data sharing between researchers and providers is not generally endorsed as standard practice, and many studies incorporating PROs do not provide direct access for children, families, or providers to research data [37]. We find that a closed model is not ideally suited for PROs collected for care-based studies. The model permits children and families to contribute valuable evidence to improve care, but risks exacerbating the therapeutic misconception, does not promote respect for persons and their privacy data, and excludes opportunities for child and family engagement.

### Model 2: child and family decision to share data

In Model 2, children and families are able to access their PRO data collected for research, are informed about their options to share these data with providers, and are shown how to access support if they have concerns. Similar approaches have been described in situations where PRO data are being integrated into electronic health records [22]. In accordance with existing guidance discussed above, researchers are responsible for identifying and alerting providers about any responses that warrant immediate clinical action, if applicable [25]. In Model 2, researchers are responsible for providing support, or at least access to materials that explain what PRO results mean in a general sense, and how these might be relevant to clinical care. However, it is emphasized that PROs will not be shared with the health care team and the decision and responsibility to share them remains with the child and family.

This model has several advantages. It promotes respect for persons since decisions about whether to share PROs submitted for research beyond the research team reside with children and families. This model also contributes to the protection of data privacy and confidentiality insofar as it limits data sharing without the express wishes and knowledge of a participant. This model may help to promote respect for children’s developing autonomy and understanding of their own health [38], aligns with control over their own data, [4] and supports shared decision making, which may be desirable to children and families and is associated with improvements in children’s health, self-esteem, and care satisfaction [39–42]. As children age, this model may offer more flexibility around how they consent to share data with providers, especially if parents and children have different perspectives. This model also supports opportunities for child and family engagement; it would be vital to consider the perspectives of children and families on how this model impacts their responsibilities, and whether this is desirable. Children and families require age-appropriate information about the purpose and nature of PRO collection and space to consider their own preferences for providing or sharing PROs, including opting out.

Despite these advantages, Model 2 faces challenges. One difficulty is that it is unlikely to prevent and may exacerbate the therapeutic misconception. Research participants bear the full responsibility of understanding that PROs collected for research will not be accessible to providers by default, which requires a degree of conceptual separation between research and care activities that may be overly optimistic given the pervasiveness of the therapeutic misconception. Given that children and families must actively decide to share PRO data with their providers in this model, the greatest responsibility is placed on them to access, interpret, and integrate these data into care, which may present a significant burden on families already navigating complex health systems. Ensuring that the interpretation of PROs is clear and accessible is a critical responsibility of researchers to maintain a patient-centred approach, especially for measures where scoring or analysis is more complex [9, 34]. Families of children who independently provide PRO measures may have interest in reviewing them once complete, and this model provides an opportunity to explore this option if desired, but would require additional considerations when obtaining informed consent from children.

Model 2 faces more logistical complexities than Model 1. Once research participants have made a decision to share PRO data with the clinical team or store them in electronic health records, researchers are required to consider how and whether to support mechanisms for sharing data. Model 2 may be desirable to researchers because the need to coordinate data sharing with providers is avoided, and considerable resources (i.e., time spent developing and implementing agreements, training clinical staff) are conserved. However, as this approach does not require training for providers to interpret, discuss, and document PROs, there is potential for disconnect between children and families and informed care responses, or misinterpretation of results. If children and families wish to integrate PROs into care, it may be difficult for providers to store and track these data over time without a structure in place to do so, or find time to review the PROs during health care visits. Children and families may be disappointed if providers do not have these capacities when approached, and this could negatively impact the provider-child and family relationship.

This model offers more opportunities for child and family engagement than Model 1 but presents significant challenges for patient-centred care and research. We heard from patient and family partners that they are concerned about bearing the responsibility of initiating data sharing, putting the onus on them to manage PROs (e.g., tracking, sharing), and forgetting about or losing track of PROs. While the model has the value of empowering parents and children (consistent with their developing capacity) with complete responsibility for decisions about sharing PRO data, this must also be recognized as a challenge since it generates burdens for patients with interests in their data being shared, but limited time or capacity to promote these interests. This approach has potential to disservice children and families with less time and resources, and limited ability to advocate for themselves [43].

### Model 3: child and family option to share data with clinician opt-in/opt-out

In Model 3, the decision to share PRO data with providers or in health records remains with research participants, but there is an additional step where providers are contacted by researchers and can provide consent to review PRO data, if children and families also consent to this. During informed consent, researchers are required to clearly communicate to children and families that PROs have the potential to be integrated into care, but only when both they and their health care provider(s) opt in.

There are challenges associated with this model, including logistical constraints. Children and families are required to identify relevant providers for researchers to obtain consent from, which may be challenging given the high number of providers who can be involved in caring for children, especially those with medical complexity. In care settings where children do not have one designated provider, this model presents challenges for patient-provider relationships that are less sustained. Further, researchers would be required to identify any changes in providers that occur during a study and obtain consent with new providers as necessary. To reduce the burden of seeking consent from providers to review and discuss PROs for each of their patients who participate in research, researchers may consider asking providers to opt in for all their patients enrolled in the study, should their decision be driven by a broad approach rather than individual patient characteristics. Researchers also assume a significant responsibility to ensure data collection and sharing are feasible (including between institutions), secure, and timely, and providers are trained to evaluate, interpret, and respond to PROs. In turn, providers assume an extra responsibility to review, document, and discuss PROs, if they opt in. This may present challenges around research sponsor or institutional policies that may already be in place to protect patient privacy, and require safeguards to distinguish research from clinical data in both patient health records and care responsibilities for providers. For this model to be successful, children and families need clear information about the applicability of PROs to their care (i.e., not all PROs will have direct or immediate impacts), a realistic timeline of when PROs may be discussed with them, where PRO data will be stored, such as in an electronic health record, and how they will be transferred, particularly if shared with providers at other institutions. This model is not as accommodating as Model 2 for a child or family’s changing preferences about data sharing, because consent would need to be withdrawn instead of simply choosing not to share them at the next encounter. This model also raises the possibility that a provider does not consent to receive PRO data from a child, which may introduce an uncomfortable dynamic to their relationship and limits the potential for incorporating PROs into care. However, support for measuring PROs from health care systems, funding bodies, journals, and patient groups may facilitate uptake [10]. This highlights the importance of child and family engagement in health care decision-making; if children and families have opportunities to indicate their preferences about PRO sharing, providers may be more likely to opt in. Nonetheless, the possibility of providers opting out should be discussed with participants to inform their decision-making.

Notwithstanding these challenges, Model 3 respects patient autonomy. Similar to Model 2, Model 3 allows children and families to have the opportunity to be in control of which data they share with providers or family members when children provide PROs independently, and this may support patient empowerment [38], incorporating overlooked areas of well-being by using PRO data during care [4], and preferences for data privacy. This can also be beneficial for children, where PROs collected for research that reflect parent and family (rather than child) experiences and have less bearing on children’s clinical care can be purposely omitted from health care intended to be focused on children. This model offers the opportunity for providers to help explain PRO results, rather than leaving children and families to their own devices. For example, a PRO might reveal clinically meaningful information that could lead to a diagnostic process or identify an area in which a child is outside of the population norm on a particular area of development. A provider who opts in to receive the data would be well-positioned to explain these potential outcomes. When both parties opt to share PRO data, this model presents the lowest risk of the therapeutic misconception because providers are in the best position to respond to children’s PRO responses. Though Model 2 presents an opportunity for children and families to engage with their own research data and integrate them into care, Model 3 can encourage children, families, and providers to make intentional partnerships around using research data to a higher potential, and reduce research waste.

Overall, our preference is for Model 3. The model has challenges, such as the possibility of tension if children and families opt-in but providers do not, efficiency and implementation complexities, and additional requests being made of already heavily burdened providers and researchers. However, the support for dialogue and shared decision-making about PROs between children, families, and providers in this model and strengths in patient empowerment offer advantages that meet the ethical criteria outlined. This model provides the opportunity for clinicians to opt-in or refuse to receive PRO data, which increases the likelihood that this information will be received and reviewed rather than incorporated into health records without a clear plan. Model 3 also shifts the obligation of promoting next steps for data sharing from patients to a shared arrangement between children, families, researchers, and clinicians. For these reasons, Model 3 seems best equipped to promote beneficence and contribute to children and families’ overall well-being. The most desirable model is one that considers the perspectives, preferences, and needs of children, families, researchers, and providers.

## Conclusion

As a multi-disciplinary group of researchers, providers, patient and family partners, and ethicists, we analyzed 3 models for handling PRO data collected in care-based research studies and conclude that there are ethical advantages to share PROs with children, families, and providers. Though PRO sharing models that account for the interests of children, families, providers, and researchers will look different across various clinical and research contexts, the ideal approach is one that prioritizes transparency, respect for persons, and engagement with children and families.
